# Asymptomatic Ileal Intussusception: A Rare Twist in a Marginal B-cell Lymphoma

**DOI:** 10.7759/cureus.82267

**Published:** 2025-04-14

**Authors:** Marco A Hernández Guedea, Adriana M Guajardo-Montemayor, Luis A González Torres, Juan A Martínez-Segura, Gerardo E Muñoz-Maldonado

**Affiliations:** 1 General Surgery, Hospital Universitario Dr. José Eleuterio González, Universidad Autónoma de Nuevo León, Monterrey, MEX; 2 Internal Medicine/Gastroenterology and Digestive Endoscopy, Hospital Universitario Dr. José Eleuterio González, Universidad Autónoma de Nuevo León, Monterrey, MEX; 3 Gastroenterology and Digestive Endoscopy, Hospital Universitario Dr. José Eleuterio González, Universidad Autónoma de Nuevo León, Monterrey, MEX

**Keywords:** marginal cell lymphoma, mucosa-associated lymphoid tissue (malt) lymphoma, screening colonoscopy, small-bowel intussusception, ‘surgical resection’

## Abstract

A 75-year-old male patient with a history of type 2 diabetes, hypertension, interstitial pneumonitis, and gastroesophageal reflux disease underwent a screening colonoscopy that revealed a 3-4 cm ulcerated ileal tumor. Subsequent diagnostic evaluations, including endoscopy and contrast-enhanced CT, identified additional findings of chronic atrophic gastritis with *Helicobacter pylori* and ileocecal intussusception. Histopathological analysis confirmed a marginal zone B-cell lymphoma with CD20+ expression and negative surgical margins following a right hemicolectomy. *H. pylori* eradication was successful, and the patient remains asymptomatic with no evidence of recurrence. It can present atypically, including intussusception. Early detection and appropriate management are crucial for achieving favorable outcomes.

## Introduction

Marginal zone B-cell lymphoma (MZL), a type of non-Hodgkin lymphoma (NHL), ranks as the second most common indolent lymphoma, comprising 7% of NHL cases. MZL has three subtypes: mucosa-associated lymphoid tissue (MALT) lymphoma (70%), splenic lymphoma (20%), and nodal lymphoma (10%) [1]. MALT lymphoma predominantly develops in the stomach and is associated with an infection of *Helicobacter pylori*. *H. pylori* infection induces chronic gastritis by activating specific T-helper cells, leading to polyclonal B-cell expansion, a crucial factor in carcinogenesis [2,3]. While rare, MZL-MALT can also occur in the small intestine. Intussusception, a condition in which one segment of the bowel telescopes into an adjacent segment, occurs infrequently in adults [4]. In adults, malignancies contribute to 32.9% of intussusception cases, benign pathologies to 37.4%, and idiopathic causes to 15.1% [5]. We present the first documented case, to the best of our knowledge, of small intestinal MZL-MALT presenting as asymptomatic intussusception.

## Case presentation

**Figure 1:**
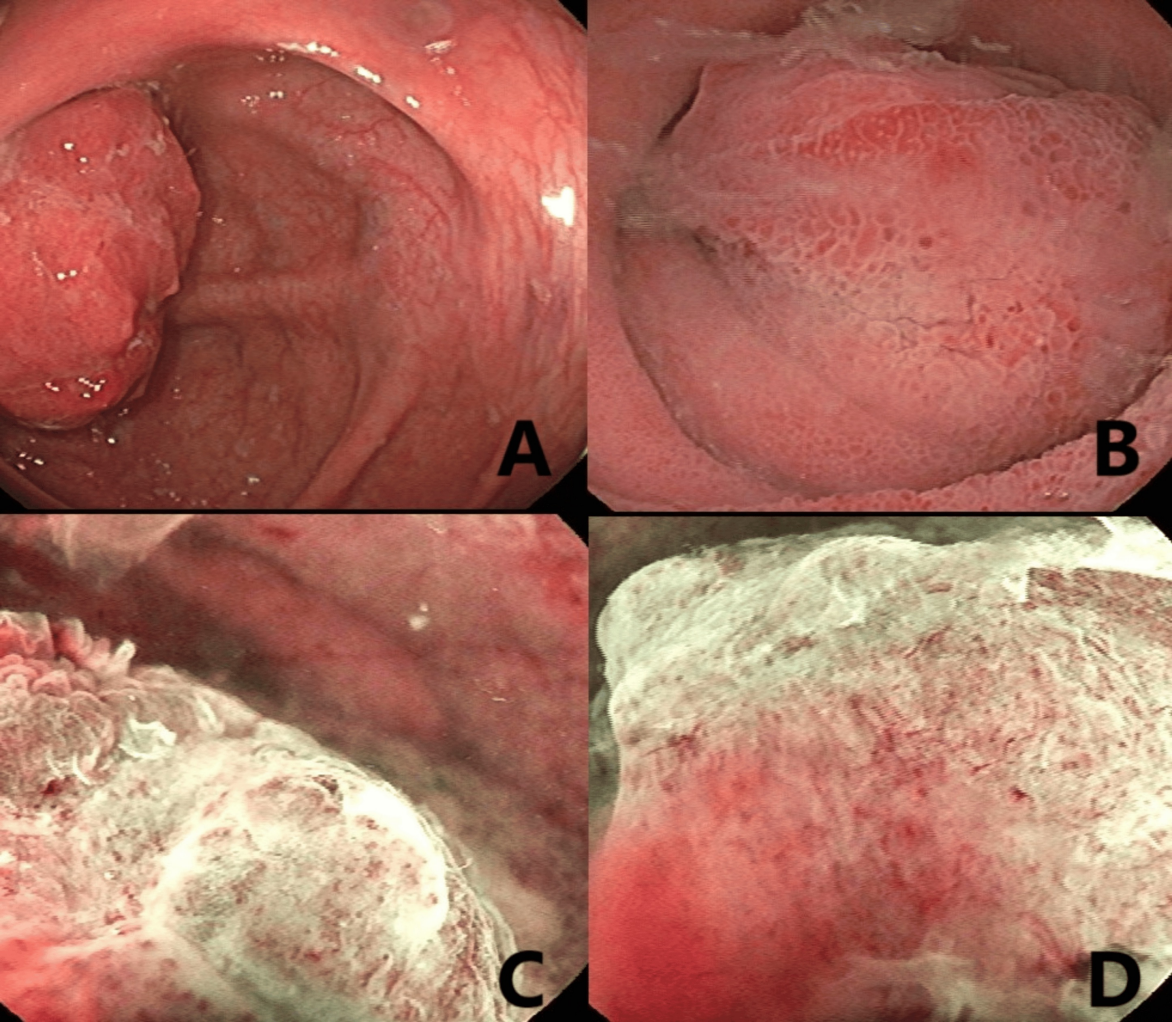
Colonoscopy findings (A-B) Colonoscopy images of the ileocecal tumor using white light endoscopy; (C-D) Narrow band imaging of the lesion, showing significant distortion in the vascular pattern and loss of surface integrity.

A 75-year-old male patient with a history of longstanding type 2 diabetes mellitus, hypertension, interstitial pneumonitis, gastroesophageal reflux disease, a 30-year smoking history, and previous inguinal hernia repair presented to the gastroenterology clinic for a screening colonoscopy. During the procedure, we identified a 3-4 cm ulcerated tumor in the terminal ileum (Figure 1).

**Figure 2:**
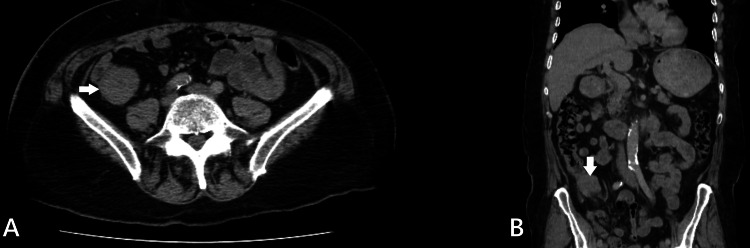
Abdominal computed tomography findings (A) The axial plane of the abdominal CT scan shows a focal thickening of the ileocecal valve associated with fat stranding and no perilesional lymph nodes (white arrow); (B) The coronal plane of the abdominal CT scan shows the same finding (white arrow).

**Figure 3:**
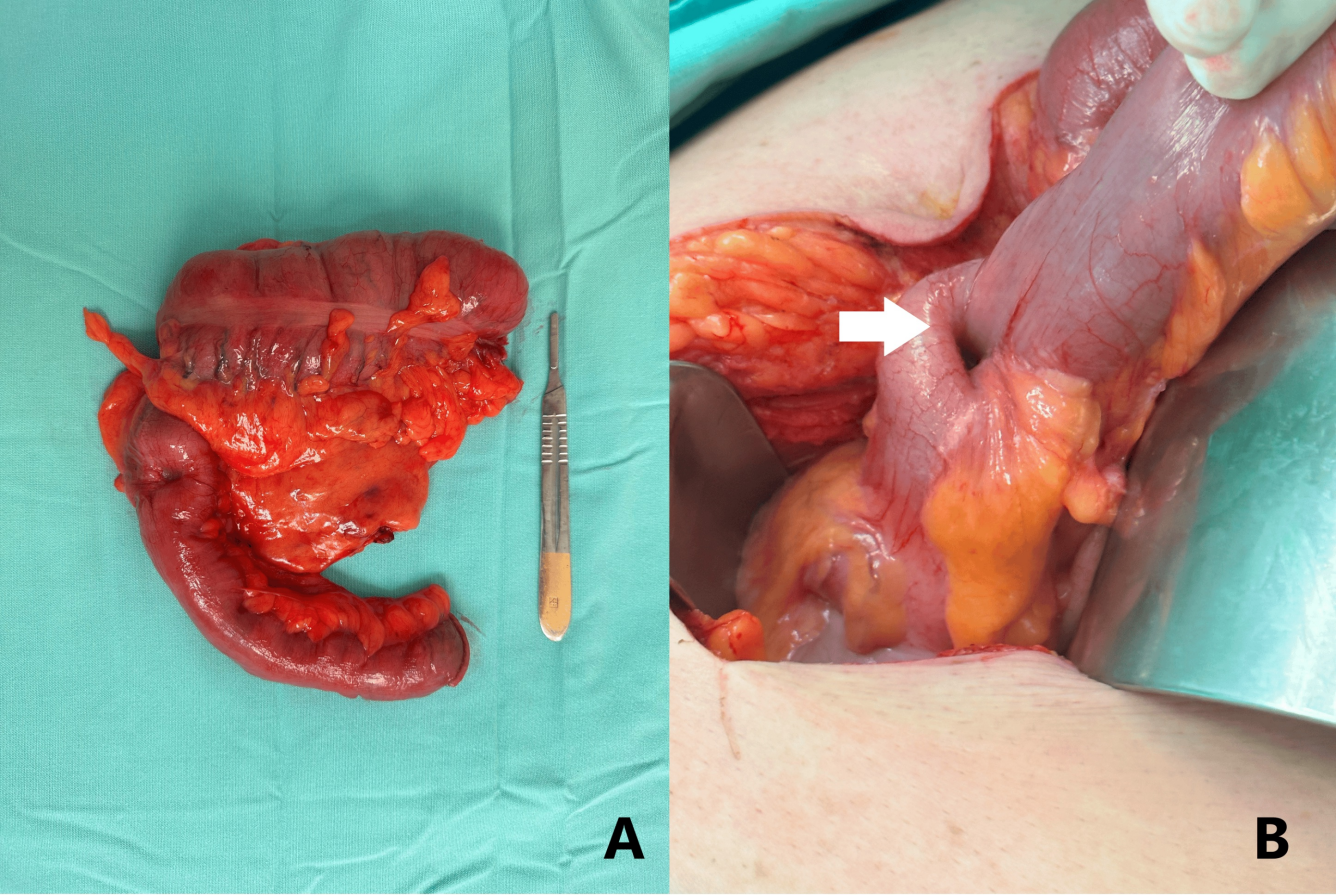
Surgical images (A) Surgical image after the resection of the intussuscepted intestinal loop; (B) Ileocecal Intussusception during the surgical intervention, white arrow shows the intussusception site.

We performed fine-needle aspiration (FNA) and obtained mucosal biopsies using forceps. A thorax and abdomen CT scan showed focal wall thickening of the ileocecal valve with associated fat stranding but no perilesional lymph nodes (Figure 2). Despite the lack of lymphadenopathy, our high suspicion of malignancy led us to proceed with an open right hemicolectomy and ileum-to-transverse colon anastomosis. During the procedure, we observed an ileocecal intussusception (Figure 3).

**Figure 4:**
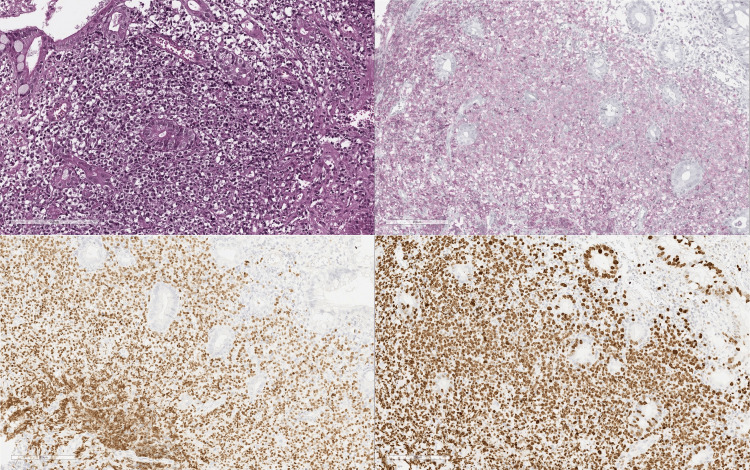
Endoscopic pathology findings (A) Hematoxylin and eosin (H&E) staining reveals a diffuse lymphoid infiltrate composed of monocytoid-appearing cells with clear cytoplasm, centrocyte-like cells, and a few large cells; (B) Immunohistochemical staining for BCL2 shows that most infiltrating cells are positive, displaying a poorly defined nodular pattern in the lamina propria; (C) PAX5 immunohistochemistry highlights most infiltrating cells as positive, showing a poorly defined nodular pattern in the lamina propria; (D) KI67 staining is positive in 90% of the neoplastic cells, indicating a high proliferation index. Additionally, immunohistochemistry for CD3 and INSM1 was negative.

Histopathological analysis of the endoscopic biopsies revealed chronic gastritis in all affected segments of the stomach, along with the presence of *H. pylori*. Biopsies taken during the colonoscopy confirmed the diagnosis of NHL, which tested positive for BCL2 and PAX5 (Figure 4). The histopathological examination of the surgical specimen identified CD20+ MZL with negative surgical margins. During follow-up, the patient remained asymptomatic after successful eradication of *H. pylori*, with no signs of recurrence or extra-intestinal disease observed after one year.

## Discussion

The WHO classification identifies four types of MZL: extranodal MALT, primary cutaneous, nodal, and pediatric [6]. Extranodal MZL (EMZL) frequently occurs in the gastrointestinal tract, with approximately 85% of cases involving the stomach, followed by the small intestine and colon [7]. The prevalence of small-intestine lymphoma (SIL) varies across studies, ranging from 8.6% to 32.4% [8,9]. In a study of 80 patients with SIL, the ileum was the most commonly affected site, accounting for 46% of cases, followed by the jejunum (16%), ileocecal region (11%), duodenum (5%), and multiple sites (6%) [10]. MZL was responsible for 19% of these cases.

The male-to-female ratio for SIL with MZL is 1.5:1, indicating a male predominance. The median age at diagnosis is 59 years, and up to 90% of patients present with gastrointestinal symptoms, including abdominal pain, obstructive symptoms, melena/hematochezia, weight loss, acute diarrhea, and protein-losing enteropathy. Approximately 9.6% of patients may remain asymptomatic [11]. Small bowel intussusception is uncommon in adults, with malignancies accounting for up to 40% of cases; however, it remains a rare manifestation of MZL. A review of the literature revealed four other cases of MZL associated with intussusception (Table 1). Primary diagnostic methods for MZL include bone marrow biopsy, 2-fluorodeoxyglucose F 18 (18F-FDG)-positron emission tomography (PET)/CT imaging, and surgical removal of affected lymph nodes [11]. In our case, we used colonoscopy and imaging techniques to establish the diagnosis.

**Table 1:** Case reports presenting small intestine marginal zone B-cell lymphoma and intussusception. NS: not specified; R-CHOP: rituximab, cyclophosphamide, doxorubicin hydrochloride, vincristine sulfate, and prednisone

Author	Age of patient	Sex	Symptoms	Helicobacter Pylori test	Diagnosis	Location	Treatment
Murino et al., 2012 [12]	58	Male	Abdominal pain (associated with eating), Weight loss	-	Double-Balloon Enteroscopy	Jejunum	R-CHOP (3 cycles)
Adams et al., 2016 [7]	83	Female	Abdominal pain (associated with eating), Diarrhea, Abdominal distension	NS	Laparotomy	Terminal ileum	NS
Coelho et al., 2024 [4]	78	Male	Abdominal pain (relieved by passage of loose stool), Vomiting, Weight loss, Abdominal distention	NS	Laparotomy	Ileum	R-CHOP (6 cycles)
Da et al., 2024 [13]	32	Male	Abdominal pain	NS	Colonoscopy	Ileum	Right Hemicolectomy + Lymph node dissection R-CHOP (4 cycles)

Treatment options for MZL remain uncertain. There is insufficient quality data on small intestine EMZL management. Still, the basic recommendation for intestinal sites is that in stage I, patient cases should be individualized and have an excellent prognosis (10.6% deaths at 15 years of colonic EMZL) [1]; advanced stages are considered incurable, and chemotherapy is the standard of care (Bendamustine and Rituximab) [1]. 

Markopoulos et al. reported a similar case [11]. They included a scoping review describing the combined experience of 53 cases of small intestine EMZL, where they found that 37.5% of cases opted for surgery and chemotherapy, 33.3% for surgery, and 25% for chemotherapy alone. Chemotherapy protocols most often included rituximab, cyclophosphamide, hydroxydaunorubicin, oncovin, and prednisone (R-CHOP) or rituximab, cyclophosphamide, vincristine, and prednisone (R-CVP) immunochemotherapy. After a median follow-up of 15 months, 82% of patients achieved clinical remission, 3% of patients had disease progression, and 11.7% died during follow-up. Among the deaths, they attributed 50% to disease progression, with two of the four MALT lymphoma-related deaths occurring in patients with advanced-stage disease at diagnosis. We staged the present case as an Ann Arbor stage I and opted for surgical resection without chemotherapy. The patient showed no signs of disease recurrence after a follow-up with a PET and a contrasted CT scan (after seven months). Timely diagnosis played a key role in this favorable outcome. 

The primary strengths of this case report lie in its unique presentation, diagnosis, and treatment approach. Our experience offers valuable insights into MZL in the small intestine. However, a notable limitation is the absence of complete histopathological imaging evidence.

## Conclusions

This case highlights a rare presentation of small intestine MZL-MALT as asymptomatic intussusception. Early detection through endoscopy and imaging enabled successful surgical resection, with no recurrence observed after one year. In the absence of standardized treatment guidelines, individualized management remains essential. This report adds to the limited literature on MZL-MALT and underscores the need for further research.
